# Pilot Cluster Randomized Controlled Trial of Integrative Nutritional Counseling Versus Standard Diabetes Self-Management Education for Chinese Americans with Type 2 Diabetes

**DOI:** 10.1089/heq.2020.0002

**Published:** 2020-10-02

**Authors:** Evelyn Y. Ho, Sunny Pak, Genevieve Leung, Shuwen Xu, Choi Kwun Yu, Frederick M. Hecht, Jane Jih, Maria T. Chao

**Affiliations:** ^1^ Department of Communication Studies, University of San Francisco, San Francisco, California, USA.; ^2^ Asian American Research Center on Health, San Francisco, California, USA.; ^3^ Chinatown Public Health Center, San Francisco, California, USA.; ^4^ Department of Rhetoric and Language, University of San Francisco, San Francisco, California, USA.; ^5^ School of Nursing and Health Professions, University of San Francisco, San Francisco, California, USA.; ^6^ Osher Center for Integrative Medicine, University of California San Francisco, San Francisco, California, USA.; ^7^ Division of General Internal Medicine, Department of Medicine, University of California San Francisco, San Francisco, California, USA.; ^8^ Multiethnic Health Equity Research Center, University of California San Francisco, San Francisco, California, USA.

**Keywords:** type 2 diabetes, Chinese medicine, Chinese Americans, integrative medicine, nutrition

## Abstract

**Purpose:** Chinese Americans (CAs) with diabetes and limited English proficiency often struggle to adhere to standard diabetes diets focused on food measurement/restriction. Chinese medicine principles commonly inform food choices among CAs but are rarely acknowledged in nutritional interventions. We developed and tested feasibility of a theoretically informed integrative nutritional counseling (INC) program that combines Chinese medicine principles with biomedical nutrition standards.

**Methods:** We randomized diabetes self-management education (DSME) classes to include either: (1) usual nutrition curriculum based on American Diabetes Association (ADA) recommendations delivered by a diabetes educator (control) or (2) INC curriculum based on a combination of ADA recommendations and Chinese medicine principles delivered by a diabetes educator and a licensed acupuncturist (intervention). All DSME enrollees were invited to participate in research entailing data collection at three time points: baseline, after the DSME nutrition class, and at 6-month follow-up. Using validated measures, we collected dietary self-efficacy, diabetes distress, diet satisfaction, and dietary adherence. We also measured weight and glycemic control.

**Results:** Study participants were 18 Cantonese-speaking patients with diabetes who were predominantly female and older, with low levels of income and acculturation. Intervention and control groups were similar at baseline. INC performed similarly to usual DSME with 100% of participants reporting the INC booklet helped their learning. Dietary adherence significantly improved in participants who received the INC curriculum.

**Conclusion:** INC is feasible to implement as part of DSME classes and shows promise as a complementary culturally sensitive addition to usual diabetes nutrition education for CA patients.

## Introduction

Chinese Americans (CAs) are at a higher risk for diabetes than European-descent Americans with similar body mass index.^1,2^ Given the daily demands of diabetes management, language barriers are an important source of stress and anxiety for CAs.^3^ Less acculturated, Chinese-speaking CAs with diabetes have lower diabetes knowledge, health, quality of life,^4^ and quality of care,^5^ worse diabetes self-management,^6^ and higher hemoglobin A1C (HbA1c).^2^

Health research with ethnic minorities emphasizes the importance of culture. However, too often in health interventions, culture is addressed merely on a surface level through appropriate language and/or ethnic pictures.^7^ While health communication efforts targeting CAs with diabetes need to use Chinese language^3,8^ and pictures of Chinese people and foods,^7^ a deeper, and more equitable recognition of culture acknowledges how people understand the causes and treatments of illnesses.^9^ Chinese medicine beliefs and practices are highly prevalent and deeply rooted in CA communities.^8,10,11^ Many CAs find culturally targeted diabetes materials to be overly simplistic^3^ and do not address their use of Chinese medicine^12–15^ or its principles such as balancing foods with hot and cold properties.^16^ Because minimal research or clinical practice has integrated these beliefs for promoting self-management and healthy dietary practices, an already underserved population is further disempowered.

**FIG. 1.**
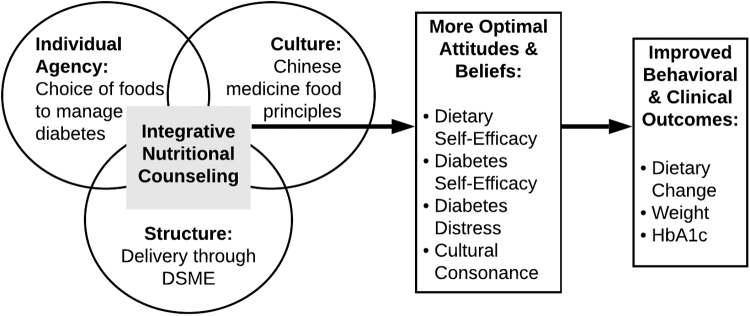
INC Framework to Improve Health Outcomes INC is an educational curriculum that accounts for patient's individual agency, culture, and structural factors that may improve patient attitudes and beliefs about type 2 diabetes, which can lead to improved behavioral and clinical outcomes. INC, Integrative Nutritional Counseling.

To address this gap, we developed integrative nutritional counseling (INC) using a Culture-Centered Approach (CCA), a theory which recognizes how individuals are embedded in larger cultural contexts and structures that affect their ability to participate in health.^17^ CCA projects begin by acknowledging participants' positive health practices even if those practices are not recognized as formal health care. CCA theorizes that positive health communication occurs when accounting for individual agency, culture, and structure (Fig. 1). INC was developed to closely align with how CAs eat and think about food and health (agency) by incorporating principles from Chinese medicine (culture) while meeting current biomedical nutrition standards, including through diabetes self-management education (DSME) delivery (structure).^18^ INC is designed to meet people's health belief systems, acknowledge currently efficacious practices and beliefs, and promote healthy behaviors and beliefs.

The few studies of culturally targeted diabetes education for CAs suggest improvements in patients' self-efficacy, diabetes knowledge, quality of life, distress^19^ and glucose control.^8,20^ However, the length of prior interventions (6–24 weeks) present significant logistical barriers and substantial time commitments for participants. In an initial pilot, we tested INC as a 2-h intervention taught by a diabetes educator at university campuses. We learned that participants wanted to learn directly from a Chinese medicine expert and that offering the intervention in nonclinical settings limited access to motivated, highly educated participants. In response, we adapted INC to be cotaught by a nurse educator and Chinese medicine practitioner as part of patient education provided in a primary care clinic. This article presents feasibility and acceptability of delivering INC as part of existing clinical services and findings from a small cluster, randomized controlled trial comparing INC with usual diabetes self-management education (DSME) for Cantonese-speaking patients with diabetes.

## Methods

### Research design

**FIG. 2.**
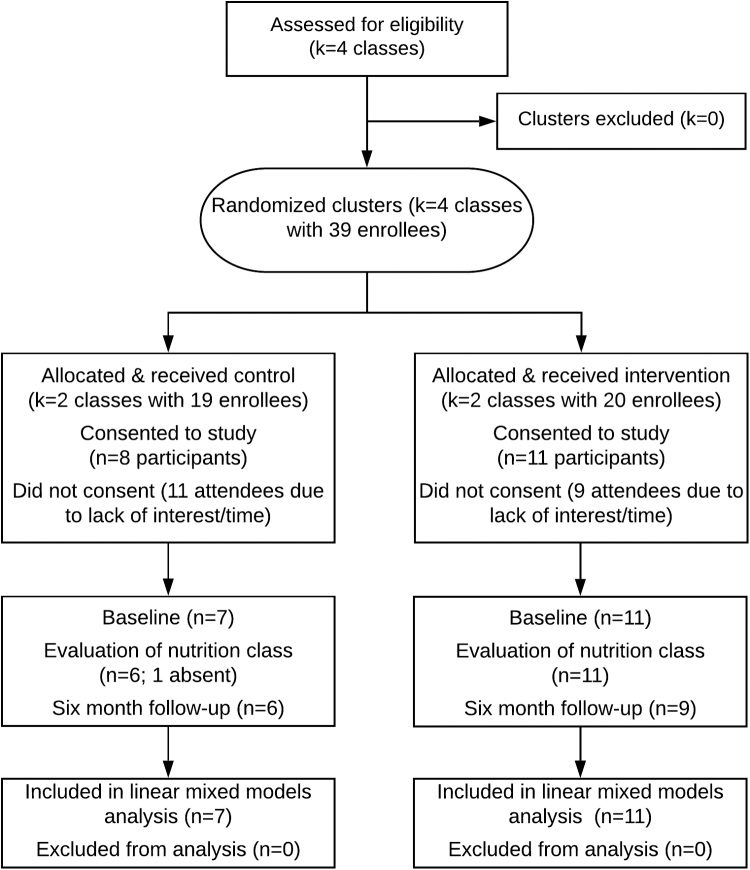
CONSORT diagram.

We conducted a pilot, two-arm cluster randomized controlled trial (Fig. 2) to compare usual diabetes education versus diabetes education with INC. DSME classes in a primary care clinic were randomized to include either a usual nutritional curriculum (DSME) or INC curriculum (DSME+INC). The cluster randomized design allowed us to assess the feasibility of INC implemented through existing services and the potential for clinical uptake of the intervention. The study was approved by University of San Francisco and University of California San Francisco Institutional Review Boards.

### Setting

Our study was conducted at the Chinatown Public Health Center (CPHC) of the San Francisco Health Network, a clinic that provides primary care, dental, community health education, nutrition, and outreach to over 5000 patients annually. CPHC has served the Chinatown community since 1929, offering language services in English, Cantonese, Mandarin, Tagalog, and Vietnamese. Over 80% of CPHC patients speak Cantonese as their primary language, most patients are low income, with high school or lower education, and diabetes is the fourth most common diagnosis for patient visits. CPHC offers group DSME quarterly by two registered nurses (RNs) who also provide direct clinical services.

### Participants

Eligible participants were fluent Cantonese-speaking/reading CAs, ≥21 years old, self-reported type 2 diabetes or prediabetes, and enrolled in a DSME class at CPHC. All patients enrolled in DSME during the study period received a telephone or in-person invitation to participate in a study to improve diabetes self-management. Interested CAs were screened by bicultural, Cantonese-speaking research assistants. Study participants were blind to the intervention assignment because nutrition classes were implemented as part of routine services offered. Consented participants completed baseline interviews before DSME classes, a brief survey in the middle of DSME classes, and a final interview 6 months after classes.

### Study procedures

The control group of usual DSME included three 2-h-long classes in Cantonese, held once weekly. Before class, participants met with research staff for surveys, a 30-min semistructured interview regarding their experience with diabetes, weight, and HbA1c. The first class covered basics, definitions of diabetes, and exercise. The second class covered diabetes diet mainly focusing on identifying and counting carbohydrates and introducing the glucometer. In the third class, participants learned about diabetes medications and created individual self-management plans.

Intervention group (DSME+INC) participants completed data collection and received DSME similar to the control group with the following modifications. Participants met with an acupuncturist at baseline for up to 30 min instead of a research assistant. The acupuncturist assessed each participant using diagnostic techniques typical of traditional Chinese medicine, including tongue and pulse exam and clinical questions about signs and symptoms, then categorized participants into one of three INC assignments (described below). At the second DSME class about nutrition, intervention participants were given their Chinese medicine diagnosis and learned about nutrition for their INC assignment. This class was cotaught by RN diabetes educators and the acupuncturist who did the diagnosis. The three educators' preparation and coordination for the intervention (total 2 h) was not perceived as burdensome and they reported that the process was reasonable to deliver as a team.

We developed INC with input from biomedical providers, Chinese medicine practitioners, and CAs with type 2 diabetes.^18,21^ INC includes a 44-page color guidebook in English/Chinese and corresponding curriculum for a 2-h class (www.INCguide.org). INC recommendations reinforce biomedical nutrition standards (e.g., distributing carbohydrates throughout the day and using a MyPlate method: one-quarter carbohydrate-rich foods, one-quarter protein, and half vegetables^22^) and culturally specific practical tips (e.g., recognizing that dumplings are both carbohydrate *and* protein).

A key innovation of INC is the integration of Chinese medicinal food principles. From a Chinese medicine standpoint, each person with diabetes may exhibit different Chinese medicine diagnoses falling broadly into one of three Chinese medicine conditions: heat, dampness and heat, and weakness/coolness.^18^ Patients are diagnosed and subdivided into these basic assignments, with corresponding treatment goals to: (1) reduce heat, (2) rid damp/heat, or (3) warm. The INC guidebook contains food lists and pictures of vegetables, carbohydrates, proteins, and other foods that patients are encouraged to “eat more” and/or “eat less” corresponding with the three broad treatment goals.

### Measures

At baseline, we collected sociodemographic variables, acculturation (preferences in language, food, friends, and identification with home/new culture),^23^ and beliefs in Chinese/Western medicine (measuring beliefs about the therapeutic effects, side effects, and efficiency of both forms of medicine).^24,25^ Feasibility and acceptability of the intervention were measured through postcourse evaluation that included open- and close-ended questions such as: what was your overall experience with diabetes nutrition education; would you recommend these classes to a friend with diabetes; is there anything you would change about the classes?

Attitudes and beliefs from pre- to postintervention, were assessed through dietary self-efficacy,^26^ diabetes self-efficacy,^27–29^ and diabetes distress.^20^ Participants were asked about dietary adherence through two validated measures of food frequency: Starting the Conversation (8 items, possible range 0–16) and a culturally sensitive adaption of the Mediterranean diet score (16 items, possible range 0–80).^30^ We chose the Mediterranean diet scale because it is a validated measure of healthy eating. The scale separates refined from nonrefined grains and identifies “unhealthy” items such as full-fat dairy products and alcohol. We adapted the scale by including Chinese-specific foods, such as dumplings, fried dim sum, and sweet boba drinks. We also asked open-ended interview questions such as: What changes have you made in your diet in the last 6 months? Have you incorporated any of the diet suggestions into your everyday lives? Have the dietary changes you have made been difficult? Weight was measured using the same scale and glycemic control was measured by HbA1c.

### Data analysis

Baseline characteristics of participants, including sociodemographics and health condition variables, in each arm were compared to assess group equivalency. For categorical measures, differences were assessed by Fisher's exact test. Linear mixed models (LMMs) were used to test significance of change in means in dietary self-efficacy, dietary adherence, weight, HbA1c, and other outcomes of interest. LMMs included random effects for persons nested within clusters and fixed effects of treatment group (DSME vs. DSME+INC), time, and group–time interaction. Quantitative analysis was conducted with Stata Statistical Software 16.0.^31^

Qualitative interviews were transcribed/translated by bilingual/bicultural research assistants who conducted the interviews. Using ATLAS.ti 8.3.0,^32^ the first author used open, axial, and selective coding^33,34^ to identify excerpts regarding patient experiences, satisfaction, knowledge, behavioral changes, cultural congruence of the diet, and barriers to diabetes diet/management. These were then iteratively discussed with the third author to refine categories and validate themes related to the effects of DSME(+INC) on patients' attitudes, beliefs, and behavioral outcomes. They chose representative quotes that best illustrated each theme that were shared with the full research team to discuss and confirm interpretations.

## Results

### Study feasibility

Four classes at CPHC were randomized: two control (DSME) and two intervention (DSME+INC) groups. Thirty-nine patients were enrolled before and attended the first DSME session; 19 consented to study participation and 18 completed baseline surveys (7 control; 11 intervention). Primary reasons for declining study participation were lack of time or interest. We collected 6-month follow-up data from 15 participants (6 control; 9 intervention). Participants were Cantonese-speaking patients with diabetes, 83% female, 100% with income < $50,000, with an average age of 62 years, and slight preference for western medicine over Chinese medicine. Compared with control, a greater proportion of intervention participants were from mainland China and had lower levels of acculturation, although differences were not statistically significant (Table 1).

**Table 1.** Baseline Characteristics of Study Participants (*n*=18)

Characteristic	Control (N*=7), *N (%)	Intervention (N*=11), *N (%)
Age, years^a^	63.2±8.7	61.1±10.5
Sex, female	7 (100)	8 (73)
Country of origin		
Mainland China	3 (43)	11 (100)
Hong Kong	1 (14)	—
Other	3 (43)	—
Married	4 (57)	10 (91)
Education level, high school or less	6 (100)^b^	7 (64)
Employment status		
Full time	1 (14)	2 (18)
Part time	3 (43)	5 (45)
Disability	—	3 (27)
Unemployed	1 (14)	—
Retired/Other	2 (28)	1 (9)
Total income		
$30,000 or less	6 (100)^b^	10 (91)
$41,000 to $50,000	—	1 (9)
Insurance status		
Public	5 (71)	6 (55)
Private	—	3 (27)
Other	2 (29)	2 (18)
Comorbidities, mean^a^	3.6±2.9	3.6±3.5
Diabetes complications, mean^a^	1.4±2.5	1.8±2.2
Preference for Chinese medicine^a^	3.3±0.9	3.6±0.7
Preference for western medicine^a^	3.9±0.6	3.7±0.6
Suinn acculturation^a^	2.0±0.5	1.5±0.4

^a^ Data presented as mean±standard deviation.

^b^ Data based on a denominator of 6; one participant in the control group did not report their education or income.

It was feasible to include Chinese medicine dietary concepts within a biomedical clinical context. Patients assigned to DSME+INC met with the acupuncturist before the start of classes to discuss health and illness symptoms to facilitate Chinese medicine diagnoses and subsequent placement into INC assignment. Qualitative analysis demonstrated that the acupuncturist's diagnostic questions were easily answerable and tongue and pulse examination were recognizable as part of a Chinese medical framework. For example, regarding the tongue, a 61-year-old female intervention participant stated, “There are teeth imprints, right?” The acupuncturist explained that she had spleen and yin deficiency and she responded, “the quality of the Chinese yams don't seem that good.” For someone unfamiliar with Chinese medicine, this response may seem odd. However, from a Chinese medicine standpoint, eating Chinese yams is grounding and helpful for yin deficiency. While most patients did not have this level of knowledge, the notion of balancing hot, cold, and dampness was not questioned by patients.

The DSME+INC class, cotaught by the same diagnosing acupuncturist and two nurses, was well received. Participants agreed that they learned skills during the nutrition class that would improve their diabetes diet (all ratings averaged 4.0 or higher, except “I have enough money to eat this diet” and “I plan to share details of what I learned today with any other health provider”) (Table 2). Notably, 100% of the INC group reported that “Handouts helped my learning a lot” compared with 67% of control participants who received paper photocopies with American Diabetes Association (ADA) nutrition recommendations instead of the color booklet.

**Table 2.** Postcourse Evaluation of Nutrition Class

Statement	Control (N=6), mean	Intervention (N=11), mean
1. The skills I learned during this class will improve my diabetes diet.	4.5	4.5
2. I learned something new today about diabetes and diet.	4.3	4.3
3. The class prompted me to evaluate my current eating habits.	4.3	4.4
4. The diet I learned today fits my culture.	4.3	4.3
5. I feel confident that I will use the things I learned today.	4.5	4.4
6. In my next meal, I plan to use the things I learned today.	4.5	4.4
7. I feel confident that I can stick with eating healthy foods.	4.5	4.0
8. I have enough time to eat this diet.	4.5	4.1
9. I have enough money to eat this diet.	4.2	3.9
10. I have enough support from those around me to eat this diet.	4.2	4.1
11. I feel ready to use this diet next time I eat out.	4.0	4.1
12. I feel ready to use this diet next time I cook a meal.	4.3	4.3
13. I plan to share details of what I learned today with a friend.	4.3	4.4
14. I plan to share details of what I learned today with a family member.	4.5	4.2
15. I plan to share details of what I learned today with my doctor or nutritionist.	4.3	4.1
16. I plan to share details of what I learned today with any other health provider.	3.2	4.0
Handouts—helped my learning a lot (yes).	4 (67%)	11 (100%)
Instructional teaching helped my learning a lot (yes).	5 (83%)	8 (80%)^a^
Small group experience—helped my learning a lot (yes).	4 (67%)	8 (80%)^a^

Evaluation statements were rated on a Likert scale from 1 (strongly disagree) to 5 (strongly agree).

^a^ Data based on a denominator of 10; one participant in the intervention group did not complete these items.

Data collection was feasible in this population, including Chinese-translated survey measures and HbA1c draws at two time points. We experienced challenges to recruitment and retention, such as lack of transportation and inconvenient class times. Despite these barriers, patients reported appreciating the group-based classes, as a 65-year-old female (intervention group) explained “we shared our stories and information… Each class we were asked to talk about our thoughts and feelings. Then I knew what part I hadn't done enough, and I could discuss what I had done wrong and analyze it.” Additionally, a 52-year-old female participant (intervention group) appreciated the class because doctors could never spend this much time covering diabetes education with all patients.

### Effects of DSME(+INC) on knowledge, attitudes, and beliefs

We assessed changes from pre to post intervention, comparing effects of DSME with DSME+INC using validated measures. Pre to post intervention outcomes indicated greater improvements in dietary self-efficacy among DSME+INC than DSME, but these differences were not statistically significant. Nonsignificant improvements in diabetes-related distress were also observed in both groups (Table 3).

**Table 3.** Study Outcomes, Baseline, and Change at 6-Month Follow-Up

	Control, N=7		Treatment (INC), N=11	
	Baseline, mean±SE	Mean change at 6 months±SE	Baseline, mean±SE	Mean change at 6 months±SE
Attitudes and beliefs				
Diabetes self-efficacy	33.0±1.8	0.3±1.8	31.1±1.5	1.1±1.4
Bicultural self-efficacy	32.9±1.5	0.6±1.9	32.2±1.6	1.2±1.5
Family support				
Instrumental	4.0±0.3	0.3±0.3	4.3±0.2	−0.1±0.2
Emotional	3.6±0.2	−0.1±0.3	3.2±0.2	−0.2±0.3
Diabetes quality of life satisfaction	61.4±3.5	1.0±2.7	55.8±2.8	−1.8±2.2
Diabetes distress total^a^	2.1±0.3	−0.2±0.3	1.8±0.3	−0.2±0.3
Emotional burden^a^	2.7±0.5	−0.8±0.5	2.4±0.4	−0.4±0.4
Physician-related distress^a^	1.7±0.4	0.1±0.2	1.6±0.3	−0.2±0.2
Regimen-related distress^a^	2.2±0.4	−0.3±0.3	1.7±0.3	0.0±0.3
Interpersonal distress^a^	1.6±0.3	0.2±0.3	1.5±0.2	−0.3±0.3
Dietary adherence and clinical outcomes				
Starting the conversation^a^	7.1±0.7	−0.6±0.7	6.7±0.6	−0.8±0.6
Mediterranean diet^*^	54.3±3.3	−5.5±1.8	55.6±2.6	4.3±1.4
Eating self-efficacy				
Negative affect	15.0±2.9	2.5±2.8	14.3±2.3	2.7±2.3
Socially acceptable circumstances	12.7±3.7	−0.4±3.9	16.9±3.7	−0.2±3.2
Single item	3.1±0.4	0.5±0.6	3.8±0.3	0.3±0.5
Hemoglobin A1c^a^	7.5±0.4	−0.6±0.3	7.0±0.3	−0.4±0.3
Weight, lbs^a^	130.0±10.3	0.1±1.0	135.7±8.3	−0.5±0.8

^a^ Lower score indicates more optimal outcome.

^*^ *p*<0.01 for within-group changes from baseline to 6 months, and group–time interaction based on linear mixed model.

INC, integrative nutritional counseling.

From exit interviews, participants reported knowledge gained from the DSME class, including how and why diabetes happens, MyPlate proportion, carbohydrate identification, diet information, and goals for exercise (exemplary quotes: Table 4). For participants in the intervention group, feedback on what they learned about Chinese medicine was mixed. Five remembered accurately their INC type, three forgot, and one remembered inaccurately. For those who remembered, the INC information was smoothly integrated along with other diabetes education. As a 71-year-old female responded, “I check my blood glucose level. They told me to avoid certain foods. I should not eat cold foods, so I need to be aware of that. I also have to exercise frequently and regularly, as well as splitting into eating several meals a day. These are things I learned from you, if it wasn't for you, I wouldn't know.” In addition, when participants were asked whether they used Chinese medicines or medicinal foods, respondents in both intervention and control groups talked about teas, foods, and herbs used (Table 4). Integrating Chinese medicine and nutrition was relevant to this entire population.

**Table 4.** Effects of Diabetes Self-Management Education(+Integrative Nutritional Counseling): Qualitative Quotes

Themes	Representative statements (gender, age, control, or intervention [INC])
Effects on knowledge: Diabetes knowledge	Interviewer: What about diabetes patients, how can they be healthy?
	(F, 63, Control): [2 lines omitted]… Because people with diabetes do not have a normal pancreas, it does not excrete enough insulin. It will bring sugar into cells. So, diet is the first thing. Eat less per meal but eat more meals per day. And don't eat too sweet or too salty. The three low and one high. Low sugar, low oil, low salt, and high fiber. It is important.
	(M, 66, INC): I also learned that if not enough insulin is secreted from spleen, it may cause diabetes. All I can do now is to manage diabetes and prevent it from getting worse.
Effects on knowledge: Carbohydrate knowledge	(F, 66, Control): yes. So, for the plate, we split it into 4 parts. Vegetables make up most of the plate, then watch out for protein and carbs. Since I am aging now, I can't remember much and I have to always go back to it. I have a little note with me to remind myself.
	(F, 52, INC): For controlling my diabetes and also eating habits. So now I know when eating in restaurants, I can order more vegetables, less noodles. For example, noodles need to be less, vegetables should order more. It won't be like in the past when the whole bowl would be noodles. So, I know the portions, that vegetables need to be 2 portions, carbohydrates are 1 portion and fish, meat, eggs are 1 portion, for planning. Before, I wouldn't care, like to eat what I will eat.
Effects on knowledge: Specific diet/food information	(F, 62, Control): Don't eat too many sugary things. Eat brown rice. Eat less white rice. Eat more vegetables. Don't eat fruits that are too sweet, like mango and pineapple, those are sweeter. Don't say don't eat it at all, just eat less.
	(F, 65, INC): I mostly remember the eating/drinking habits. The minimum is to change. Eat less salt, less sugar. Don't eat fried food. I don't eat fried things. When I stir fry vegetables, I don't use too much oil.
	Interviewer: Do you find that using less oil makes the food less palatable?
	(F, 65, INC): Food palatability doesn't matter. Most important is its better for the body. Once I get used to it, it's fine. It's ok if it doesn't taste as good.
Effects on knowledge: Exercise	(F, 65, INC): So I learned to exercise more each day. More walking, or move more, before sleeping some kicking.
	Interviewer: So how much do you walk daily?
	(F, 65, INC): At least 30 minutes a day, most days… sometimes I just move, so that I will sweat.
Use of Chinese medicinal foods	(F, 66, Control): Yes, I drink *Wu Lou Cha* [Chinese tea] and tea that decrease sugar levels. I took some tea when I went back to Guangzhou this time. It is called *Qin Qian Lao*. If I drank this tea, it helped reduce my sugar levels.
	Interviewer: Is it like “Lou Hei Cha” [green tea]?
	(F, 66, Control): It is tea. It tastes sweet. If it is sweet, I think it is good to the spleen because sweet goes to spleen. It would make the spleen healthier, and therefore release more insulin….Once you drink it, you can check your sugar level and then you will know, it shows an effect right away. It is good.
	Interviewer: Did you use soup?
	(F, 66, Control): I put in some white mulberry bark when I cook soup. That will reduce sugar levels, too.
	(F, 61, INC): P: I prefer Chinese medicine. I rather use diet and food to maintain my health…. According to the four seasons. Because of the different weather/climate, different absorption in the body. In summer, I cannot eat foods that are too rich/nourishing, more moderate things. In winter, have to eat more nourishing heating stuff. I will separate the season.
Effects on behavior: Positive change to diet	(F, 52, Control): I eat less now. I learned not to eat until I'm really full. For each meal, I just eat until I'm 5 or 6 parts full, and I'm done. Don't eat until you're completely filled up, because that's not good for your blood sugar. So, I've become used to this way of eating until I'm 5 or 6 parts full.
	(M, 66, INC): I no longer add sugar to my coffee and realize it tastes better without sugar!
	(F, 61, INC): After the class, I found that carbs have a large effect on blood sugar…. I try to eat as little as possible. Sometimes if I ate bread, I won't eat rice. I will eat more vegetables. Because I can't eat a lot of beans. I eat more vegetables, those with less sugar, to make myself feel full.
Effects on behavior: Positive change related to INC assignment	(F, 52, INC): Yes. The TCM practitioner, she came and she told me I am considered red. So she said I need to eat more warm foods. So I follow the booklet, she has a booklet, writing what vegetables, so I try my best. She said, the snacks, I also eat, but I also put some ginger, garlic, those, anise cloves or some turmeric, I will add to it. So I hope that it will balance it out.
Effects on behavior: Positive change to exercise	(F, 52, Control): Exercise. I walk every day for about 30 minutes…. Exercising before bed every night for 5 minutes, and in the morning for 15 minutes. It's because of the class that I learned that I should do this.
	(F, 65, INC): a bit better. Because after learning from class, I reminded myself that I need to be more mindful about it. In addition, I exercise. I don't do strenuous workouts, usually just walking. I usually get off at the bus stop and walk. It's about 20–25 minutes per day. I feel pretty energized. I got better than I was 6 months ago.
Effects on behavior: Positive change to glucose monitoring	(F, 63, Control): Also, in this class, every participant got a blood glucose measuring machine. This machine is very good because I can always measure my blood sugar, such as after I had a big meal or when I don't feel okay. If I found that my blood glucose is too high on a certain day, I can reflect on my diet and make modifications to it, and I can self-manage my diabetes properly. I will usually check it before and after a meal. Two hours after a meal is when blood sugar can get really high so I will always make sure it's still in a normal range. If it's normal, then I will keep the amount of my meal and when it's too high that I will eat less.
Self-reported outcomes: Improvement	(M, 66, INC): My A1c was about 7.5 before taking the class. Then I was referred to the diabetes management class. I then walked for a few months and became more aware of my diet and food that I should consume/avoid. Therefore, I managed my diet and my A1c value dropped to 6.1. Upon attending the class, I was told to test my A1c again, the result was about the same. I tested A1c a month ago and it increased to 6.2 or 6.3. The physician recommended that I keep exercising and managing my diet. I really think I feel better than 6 months ago. I used to weigh 152–153 lbs, now I am 140 lbs. I did not know my blood sugar was high because I walked quiet often. I used to feel very hungry when walking to Fisherman's Wharf; now I realized it was something wrong with my blood sugar. I felt too hungry to walk and was dizzy, so I had to buy some bread from bakeries in Chinatown. Such situation occurred less often in the past 6 months. I also urinated less often. Not sure whether it relates to my health condition…. I used to have to urinate several times during my walk to Fisherman's Wharf. Recently, I only urinated once during the whole walk.
Self-reported outcomes: No change or uncertain	(F, 66, Control): I think it didn't change. Before I took this class, my blood sugar level, the A1c, was 6.4. Last week I checked again, it was still 6.4. So, I don't think it has a great change.
	(F, 65, INC): Because I have yet to get the blood test results from my doctor. The doctor hasn't let me see the report.
Continued challenges to diabetes self-management	(F, 66, Control): Because I go to work, having age like mine it is hard to find a job. I have no other way. Right now I'm “riding a cow to find a horse…when the horse dies go on the street and walk” ((laughs)) I have to do all sorts of things…. I know some community organizations, but at my age, first of all, it is already a failure. Secondly, if your English is not as good as those college students. So I already know who I am. I know what I'm capable of, that's just the situation. It's just like this, just living through the days. That's what I think. No other way.
	(F, 52, INC): Dietary habits yes I can manage that, but exercise is really is different. Because after going to work and getting home from work, there are a lot of other things to do. Really don't want to move. So most often it's because of time availability and also need energy and mood.

### Effects of DSME(+INC) on behavioral and clinical outcomes

We compared differences between DSME and DSME+INC at baseline and 6 months postintervention using changes in our primary behavioral outcome (dietary adherence) and key clinical outcomes for diabetes patients (weight and glycemic control). Food frequency, as measured by the adapted Mediterranean Diet score, improved among participants in DSME+INC, but worsened for DSME participants (significant group–time interaction *z*=4.26, *p*<0.001). Weight and HbA1c improved in the intervention group but were not statistically significant (Table 3).

When asked qualitatively how they were doing compared with 6 months ago (Table 4), many reported overall well-being/psychological assessments such as “feeling normal,” “happy,” “less stress,” “peaceful,” and “comfortable.” From the control group, two reported feeling better while four reported feeling about the same. In the intervention group, all nine participants with follow-up data reported feeling at least “a little” better or were waiting on HbA1c tests to make a final statement. No participant reported feeling worse than in the previous 6 months. Participants who expressed improvements reported better blood sugar measurements (both daily and HbA1c), lower weight, medication reduction, improved sleep, more exercise, and energy. Patients attributed improvements to a variety of self-reported behavior changes, including increased exercise, reducing rice, eating more vegetables, and aiming for the MyPlate proportions. From a Chinese medicine perspective, one patient reported “avoiding cold foods” which resulted in fewer leg cramps, “feeling better, and better sleep.”

### Challenges to diabetes self-management

Despite many reported improvements and benefits, participants also reported continued challenges to diabetes self-management (Table 4). From taste preferences to hunger, participants reported difficulty avoiding foods they loved especially seeing friends/family or during holidays/special occasions. Other structural challenges included balancing exercise and other healthy habits with work and other priorities. Finally, one intervention participant assigned to the warming diet was disappointed that many of the suggested foods (e.g., garlic, onions, hot peppers) were not vegetables but rather more like spices.

## Discussion

As an issue of health equity, the Chinese-speaking, low acculturated CAs in this study are at higher risk for lower quality of care^5^ and worse diabetes self-management.^6^ Meeting these patients where they are regarding health beliefs and eating practices is critical to improving diabetes self-management in this population.^3,8^ As a culturally informed Chinese language patient education intervention, INC is the first of its kind to take Chinese medically informed dietary practices seriously while maintaining a biomedical nutrition standard. Participants' preference for western over Chinese medicine is similar to previous studies, which finds that even in older, monolingual communities in the United States, CAs may prefer western medicine because it is provided through public health insurance.^21^ INC is feasible to deliver within existing community clinic settings with the main resource burden being the addition of a Chinese medicine practitioner to provide diagnoses and team teach. The coordination of care between the RNs and the Chinese medicine practitioner was not labor or time intensive and patients remarked on the benefit of having both at the educational session.

We observed similar benefits in knowledge, attitudes, and beliefs among participants in INC and in usual DSME. The one significant behavioral improvement was in the INC group's consumption of a Mediterranean diet, which encourages the large intake of vegetables, avoidance of fatty foods, and refined carbohydrates, and is especially beneficial not only for diabetes but also for cardiovascular health.^30^ However, INCs Chinese medicine-informed individual assignment may need greater clarity as it was not uniformly understood or used. For those who remembered, like previous studies of CAs with diabetes, there was evidence that they used hot/warm balance not only in making diet choices,^3,7^ but also to participate in their own health decision making.^21^ However, with just over half accurately remembering their INC type, it is not clear if the innovation of combining Chinese and western nutrition in a *personally* tailored way is what is most relevant for all participants. Instead, perhaps other components of INC were desirable. For example, participants appreciated the accompanying booklet and some asked for more to share. Future research should examine exactly what in INC is most useful.

This study is limited by small sample size and future research should recruit more participants. We were also limited by the need to randomize by course rather than individual, which further reduced our power to assess differences between intervention groups. Because we recruited patients blinded, it is unknown whether participants would have been more interested in attending DSME if they knew they would learn about Chinese medicine-informed dietary information. This study, like others that recruit for DSME in safety-net settings,^35^ had difficulty recruiting participants due to time constraints and lack of transportation. These barriers were also reported by the clinic staff as challenges to recruitment to general classes and are not specific to our study. Future studies could work with clinics to offer a greater variety of times as well as one-time classes (not requiring 3-weeks) and advertise explicitly explaining INC as integrating Chinese and western medicine. Future studies could also compare middle/high-income groups to see how traditional DSME compares with INC. With more participants, future research can quantitatively evaluate behavioral and clinical outcomes to determine if INC could serve as a useful DSME nutrition option for CAs.

## Conclusion

INC shows promise as an integrative health care educational intervention that can be delivered alongside or as part of usual DSME in clinic settings with the additional expertise of a Chinese medicine practitioner. With refinement to the intervention, INC may provide complementary, culturally sensitive education to improve self-management for CA patients with diabetes.

## Abbreviations Used

ADA: American Diabetes Association
CAs: Chinese Americans
CCA: Culture-Centered Approach
CPHC: Chinatown Public Health Center
DSME: diabetes self-management education
HbA1c: hemoglobin A1C
INC: integrative nutritional counseling program
LMMs: linear mixed models
RN: registered nurse

## References

[1] Rajpathak SN, Wylie-Rosett J High prevalence of diabetes and impaired fasting glucose among Chinese immigrants in New York City. J Immigr Minor Health. 2010;13:181–183 10.1007/s10903-010-9356-2 PMC3072239 20533090

[2] Hsu WC, Cheung S, Ong E, et al. Identification of linguistic barriers to diabetes knowledge and glycemic control in Chinese Americans with diabetes. Diabetes Care. 2006;29:415–416 16443897 10.2337/diacare.29.02.06.dc05-1915

[3] Chun KM, Chesla CA, Kwan CM “So we adapt step by step”: acculturation experiences affecting diabetes management and perceived health for Chinese American immigrants. Soc Sci Med. 2011;72:256–264 21147509 10.1016/j.socscimed.2010.11.010 PMC3046384

[4] Fisher L, Skaff MM, Chesla CA, et al. Disease management advice provided to African-American and Chinese-American patients with type 2 diabetes. Diabetes Care. 2004;27:2249–2250 15333495 10.2337/diacare.27.9.2249

[5] Choi S, Lee JA, Rush E Ethnic and language disparities in diabetes care among California residents. Ethn Dis. 2011;21:183–189 21749022

[6] Xu Y, Pan W, Liu H The role of acculturation in diabetes self-management among Chinese Americans with type 2 diabetes. Diabetes Res Clin Pract. 2011;93:363–370 21636157 10.1016/j.diabres.2011.05.010

[7] Ho EY, Tran H, Chesla CA Assessing the cultural in culturally sensitive printed patient-education materials for Chinese Americans with type 2 diabetes. Health Commun. 2015;30:39–49 24446839 10.1080/10410236.2013.835216 PMC4105327

[8] Sun AC, Tsoh JY, Saw A, Chan JL, Cheng JW Effectiveness of a culturally tailored diabetes self-management program for Chinese Americans. Diabetes Educ. 2012;38:685–694 22722610 10.1177/0145721712450922 PMC4732271

[9] Resnicow K, Soler R, Braithwaite RL, Ahluwalia JS, Butler J Cultural sensitivity in substance use prevention. J Community Psychol. 2000;28:271–290

[10] Chun KM, Chesla CA Cultural issues in disease management for Chinese Americans with type 2 diabetes. Psychol Health. 2004;19:767–785

[11] Seligman HK, Wallace AS, DeWalt DA, et al. Facilitating behavior change with low-literacy patient education materials. Am J Health Behav. 2007;31(Suppl 1):S69–S78 17931139 10.5555/ajhb.2007.31.supp.S69 PMC4286315

[12] Ahn AC, Ngo-Metzger Q, Legedza ATR, Massagli MP, Clarridge BR, Phillips RS Complementary and alternative medical therapy use among Chinese and Vietnamese Americans: prevalence, associated factors, and effects of patient-clinician communication. Am J Public Health. 2006;96:647–653 16380575 10.2105/AJPH.2004.048496 PMC1470548

[13] Kronenberg F, Cushman LF, Wade C, Kalmuss D, Chao MT Race/ethnicity and women's use of complementary and alternative medicine in the United States: results of a national survey. Am J Public Health. 2006;96:1236–1242 16735632 10.2105/AJPH.2004.047688 PMC1483863

[14] Kwan CML, Chun KM, Huang P, Chesla CA Concerns about professional Chinese medicine among Chinese immigrants with type 2 diabetes. Diabetes Spectr. 2013;26:247–253

[15] Wade C, Chao MT, Kronenberg F Medical pluralism of Chinese women living in the United States. J Immigr Minor Health. 2007;9:255–267 17431784 10.1007/s10903-007-9038-x

[16] Ho EY, Chesla CA, Chun KM Health communication with Chinese Americans about type 2 diabetes. Diabetes Educ. 2012;38:67–76 22127677 10.1177/0145721711428774

[17] Dutta MJ Communicating Health. Cambridge, England: Polity Press, 2008

[18] Ho EY, Leung G, Pritzker S, et al. Creating and pilot testing an integrative Chinese medicine diet for Chinese Americans with type 2 diabetes [Abstract]. Diabetes. 2015;64(Suppl 1):A623

[19] Chesla CA, Chun KM, Kwan CML, et al. Testing the efficacy of culturally adapted coping skills training for Chinese American immigrants with type 2 diabetes using community based participatory research. Res Nurse Health. 2013;36:359–372 10.1002/nur.21543 23606271

[20] Fisher L, Glasgow RE, Mullan JT, Skaff MM, Polonsky WH Development of a brief diabetes distress screening instrument. Ann Fam Med. 2008;6:246–252 18474888 10.1370/afm.842 PMC2384991

[21] Ho EY, Leung G, Chi H-L, et al. Integrative medicine focus groups as a source of patient agency and social change. In: *Communicating for Social Change: Intersection of Theory and Praxis.* Edited by Dutta M, Zapata D. Singapore: Palgrave-McMillan, 2019, pp. 285–310

[22] U.S. Department of Health and Human Services and U.S. Department of Agriculture. 2015–2020 Dietary Guidelines for Americans. 8th ed. December 2015. Available at https://health.gov/dietaryguidelines/2015/guidelines/appendixes (accessed 212, 2020)

[23] Suinn RM, Rickard-Figueroa K, Lew S, Vigil P The Suinn-Lew Asian Self-Identity Acculturation Scale: an initial report. Educ Psychol Meas. 1987;47:401–407

[24] Chan MF, Mok E, Wong YS, et al. Attitudes of Hong Kong Chinese to traditional Chinese medicine and Western medicine: survey and cluster analysis. Complement Ther Med. 2003;11:103–109 12801496 10.1016/s0965-2299(03)00044-x

[25] Liang C The development and examination of the Chinese-Western Medical Beliefs Scale (in Chinese version). Nurs Res. 1999;7:445–458

[26] Stotland S, Zuroff DC, Roy M Situational dieting self-efficacy and short-term regulation of eating. Appetite. 1991;17:81–90 1763914 10.1016/0195-6663(91)90063-x

[27] Coyne JC, Smith DAF Couples coping with a myocardial infarction: contextual perspective on patient self-efficacy. J Fam Psychol. 1994;8:43–54

[28] Fisher L, Chesla CA, Skaff MA, et al. Disease management status: a typology of Latino and Euro-American patients with type 2 diabetes. Behav Med. 2000;26:53–66 11147290 10.1080/08964280009595752

[29] Lorig K, Ritter PL, Villa FJ, Armas J Community-based peer-led diabetes self-management: a randomized trial. Diabetes Educ. 2009;35:641–651 19407333 10.1177/0145721709335006

[30] Panagiotakos DB, Pitsavos C, Stefanadis C Dietary patterns: a Mediterranean diet score and its relation to clinical and biological markers of cardiovascular disease risk. Nutr Metab Cardiovasc Dis. 2006;16:559–568 17126772 10.1016/j.numecd.2005.08.006

[31] *Stata Statistical Software* [computer program]. Version Release 16. College Station, TX: StataCorp LLC, 2019

[32] Atlas.ti [computer program]. Version 8.3.0. Berlin, Germany: Scientific Software Development GmbH, 2013–2018

[33] Charmaz K Constructing Grounded Theory. London: Sage, 2006

[34] Strauss A, Corbin J Basics of Qualitative Research: Techniques and Procedures for Developing Grounded Theory, 2nd ed Thousand Oaks, CA: Sage, 1998

[35] Kahn LS, Glaser K, Fox CH, Patterson A Diabetes educators in safety-net practices. Diabetes Educ. 2011;37:212–219 21357750 10.1177/0145721710397385

